# Vector Hydrophone Array Design Based on Off-Grid Compressed Sensing

**DOI:** 10.3390/s20236949

**Published:** 2020-12-04

**Authors:** Zhibo Shi, Guolong Liang, Longhao Qiu, Guangming Wan, Lei Zhao

**Affiliations:** 1 Acoustic Science and Technology Laboratory, Harbin Engineering University, Harbin 150001, China; shizhibo@hrbeu.edu.cn (Z.S.); liangguolong@hrbeu.edu.cn (G.L.); wanguangming05@hrbeu.edu.cn (G.W.); zlpasserby@hrbeu.edu.cn (L.Z.); 2 Key Laboratory of Marine Information Acquisition and Security (Harbin Engineering University), Ministry of Industry and Information Technology, Harbin 150001, China; 3 College of Underwater Acoustic Engineering, Harbin Engineering University, Harbin 150001, China; 4 Qingdao Haina Underwater Information Technology Co., Ltd., Qingdao 266500, China

**Keywords:** compressed sensing, array design, beam pattern, off-grid

## Abstract

Array design is the primary consideration for array signal processing, and sparse array design is an important and challenging task. In underwater acoustic environments, the vector hydrophone array contains more information than the scalar hydrophone array, but there are few articles focused on the design of the vector hydrophone array. The difference between the vector hydrophone array and the scalar hydrophone array is that each vector hydrophone has three or four channels. When designing a sparse vector hydrophone array, these channels need to be optimized at the same time to ensure the sparsity of the array elements’ number. To solve this problem, this paper introduced the compressed sensing (CS) theory into the vector hydrophone array design, constructed the vector hydrophone array design problem into a globally solvable optimization problem, proposed a CS-based algorithm with the L_1_ norm suitable for vector hydrophone array, and realized the simultaneous optimization of multiple channels from the same vector hydrophone. At the same time, the off-grid algorithm was added to obtain higher design accuracy. Two design examples verify the effectiveness of the proposed method. The theoretical analysis and simulation results show that compared with the conventional compressed sensing algorithm with the same aperture, the algorithm proposed in this paper used fewer vector hydrophone elements to obtain better fitting of the desired beam pattern.

## 1. Introduction

Array signal processing of underwater acoustic sources by passive sonar systems is an important topic for target monitoring, locating, and tracking. The first thing to be considered before array signal processing is the array design.

Traditional array designs often use uniform arrays; however, as the number of hardware devices increases and the aperture increases, the cost of uniform arrays also increases. Considering this problem, the design of sparse arrays has received extensive attention, and scholars from various countries have carried out a lot of exploration and proposed a series of methods. From the perspective of array structure, these methods can be divided into two major categories. The first class is the sparse selection approach [1,2,3,4,5,6], which is based on arrays that are uniformly arranged (usually array elements spaced half a wavelength apart), and a portion of the array elements are selected to be unexcited based on the optimization results. The second class is the sparse array design method [7,8,9,10,11,12,13,14,15,16,17,18,19,20], in which the array elements are randomly arranged on the array plane. The position of array elements of the sparse array design method has more freedom than the sparse selection approach, so it has a broader application prospect. At the same time, due to the highly nonlinear nature of the problem, the optimization is also more difficult.

Sparse array design can be divided into two categories based on their optimization goals. One is based on the accuracy of azimuth estimation as the objective function, i.e., using the mean-square error (MSE) between the true and estimated azimuthal angles. For a Gaussian white-noise background, the Cramer–Rao lower bound (CRLB) defines the lower bound on the mean-square error of the azimuthal estimate, which depends on the array geometry. By maximizing the CRLB, the upper bound on the mean-square error of the azimuthal estimate can be minimized to improve the accuracy of the azimuthal estimate. However, high accuracy and low sidelobe of the array response is a dilemma that is difficult to balance. Although some other constraints have been added in articles [7,8], the high false alarm probability caused by excessive side lobes is an inevitable problem. In the article [7], excessive sidelobe led to overall performance failure. Another approach is to design the array based on the optimization of the beam pattern [9,10,11,12,13,14,15,16,17,18,19,20]. The optimization of the beam pattern can obtain the signal waveform through beamforming, thereby improving the signal-to-noise ratio, obtaining the array gain, and obtaining the orientation through subsequent searches. Therefore, the second array design idea not only optimizes the beam pattern, but also brings better results for the azimuth estimation. In the second type of design ideas, a large number of works of literature use heuristic intelligent optimization algorithms to solve the optimal solution. The article [9] proposed an optimal array design for hydroacoustic planar arrays, in which array optimization was decomposed into two steps, firstly optimizing the array layout and then optimizing the array weights. The simulated annealing was used to optimize the array layout, so it had the problem of local optimal solution. The article [14] applied hybrid genetic algorithm (HGA) to the array synthesis with the array aperture and the number of array elements as the optimization objective. The article [15] proposed a modified real genetic algorithm (MGA), which took the array aperture as the optimization condition, constrained the array element spacing, and jointly optimized all the array elements to reduce the computational amount of optimization and the complexity of the model. The article [16] proposed an improved integer coding genetic algorithm (IIGA), which improved the computational efficiency of the genetic algorithm and could avoid premature convergence of the algorithm. In addition, some evolutionary algorithms have also been applied to array design, such as the differential evolution (DE) algorithm [17], the particle swarm optimization (PSO) algorithm [18], and other evolutionary algorithms. The literatures [19,20] use convex optimization to optimize the array, which can significantly save computational time. Such array design often uses heuristic algorithm to seek global optimum, such as genetic algorithm, ant colony algorithm, etc., which is theoretically optimal without the guarantee of global optimal solution, meaning the problems of such an algorithm may fall into the local optimal solution. The existing articles all have the possibility of a local optimal solution with heuristic algorithms used to find the optimal solution.

Unlike the aforementioned optimization methods, the compressed sensing (CS) method [21,22] developed in recent years has a global optimal solution. Further, compressed sensing has various advantages in terms of computational cost and flexibility, which can be applied to arbitrary arrays, i.e., arrays of any geometry and composed of elements having potentially differing radiation patterns. Therefore, CS-based methods have been applied to the design of sparse arrays [11,12,13,23,24,25]. The goal of these methods was to use as few array elements as possible while still matching the desired response exactly or almost exactly.

Compressed sensing requires a preset dictionary; that is, a gridding processing. However, gridding has a contradiction between accuracy and complexity, and requires the assumption that the optimal element position falls on the preset grid to achieve high accurate array design. In the actual application environment, the position is exactly what we need to solve. If the optimal array position does not fall on the predetermined grid, it is called grid mismatch. The phenomenon has been studied and a lot of direction of arrival (DOA) estimation algorithms based on off-grid have been proposed [26,27,28,29,30,31,32,33,34,35]. Bayesian compressed sensing has also been widely used in this field [30,31,32]. In [31], the off-grid model was solved from the perspective of sparse Bayesian learning (SBL). In [32,33,34,35,36], coprime array, one of sparse arrays, was studied using compressed sensing and the off-grid method. A novel interpolation-based DOA estimation algorithm was presented to realize the off-grid process in [34]. Compared with the grid matching model, the off-grid model not only needs to estimate the DOA when the sparse signal is obtained with a coarse grid, but also estimate the off-grid deviation parameters. A more accurate DOA estimation can be obtained by performing offset compensation on the coarse grid. Unfortunately, research on this problem has focused only on DOA estimation and other areas, with little research on grid mismatch for array design.

Additionally, in the underwater acoustic environment, the problem of array design is more complicated. There are two options for sending and receiving signals: Scalar hydrophone array (acoustic pressure hydrophone array) and vector hydrophone array. Compared with scalar hydrophone, vector hydrophone can obtain more sound field information, not only the scalar information of the sound field, but also the vector information of the vibration velocity. The application of vector hydrophone array has been studied in the articles [37,38,39,40,41,42,43,44], but there are little articles focused on the vector hydrophone array design. For the vector hydrophone array, the above-mentioned array design methods still have the following problem which needs to be solved urgently: Previous works involving CS and sparse array design tended to focus on the case of scalar hydrophone arrays; that is, the above optimization method regards each channel of the vector hydrophone array element as one optimized position. Thus, one vector array element corresponds to three optimized positions. After optimization, the three channels of a vector array element cannot be optimized simultaneously, which will not bring the optimal optimization result. Vector hydrophone arrays also face the problem of grid mismatch when using the compressed sensing method for array design. To the best of our knowledge, there are few articles on the vector hydrophone array design under the off-grid model. For this reason, the vector hydrophone array design under the off-gird model is studied in this paper. This paper introduced compressed sensing theory into the vector hydrophone array design, constructing the vector hydrophone array design problem into a globally solvable optimization problem, and proposing a CS-based algorithm with the L_1_ norm suitable for vector hydrophone arrays. The simultaneous optimization of multiple channels of the same vector hydrophone array element was realized, and the off-grid algorithm was added at the same time. Finally, an alternate iterative array design method was proposed to obtain higher design accuracy.

The rest of this paper is structured as follows. Section 2 introduces the vector hydrophone array model in detail, Section 3 describes the design method proposed in this paper in detail, Section 4 gives design examples, and Section 5 gives conclusion.

## 2. Vector Hydrophone Array Model

Compared with the scalar hydrophone array, the vector array can obtain more information at the same time. In order to simplify the model, only the sound pressure at the same point and the orthogonal two-dimensional vibration velocity $v(t)=[v_{x}(t),v_{y}(t)]$ of the vector hydrophone output were considered, where $v_{x}(t),v_{y}(t)$ respectively represent two-dimensional vibration velocity with respect to the axis x,y.

**Figure 1:**
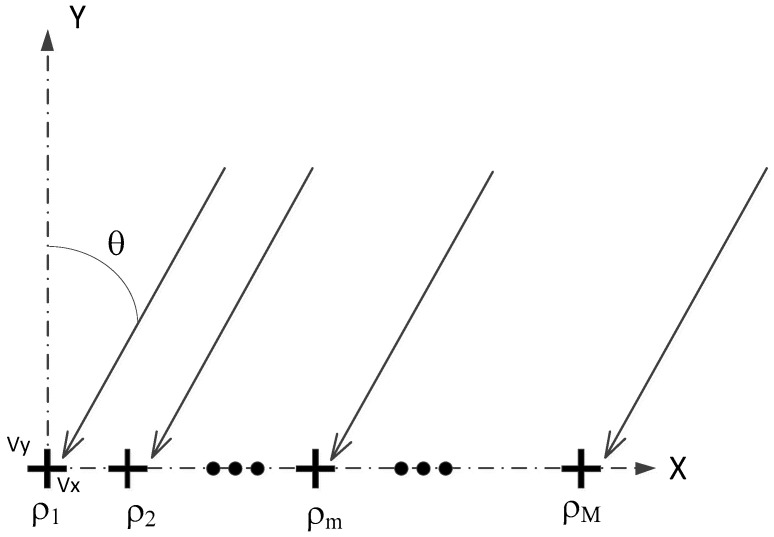
Vector hydrophone array model.

Figure 1 shows the vector hydrophone array. The symbols shown in Figure 1 are explained as follows. For two-dimensional vector hydrophone array, define the horizontal azimuth θ of the vibration velocity direction with respect to the axis x, then for an array composed of M two-dimensional vector hydrophones, the steering $a_{p}\left(\theta \right)$ vector of the received pressure signal can be expressed as follows:

(1) $$a_{p}\left(\theta \right)=\left[1,\exp\left(j\frac{2\pi \langle \rho_{m},e\rangle }{\lambda }\right),\ldots ,\exp\left(j\frac{2\pi \langle \rho_{M},e\rangle }{\lambda }\right)\right]$$

where $\rho_{m}={\left[x_{m},y_{m}\right]}^{T}$ represents the coordinate position of the first element with respect to the reference element, $e={\left[\cos\theta ,\sin\theta \right]}^{T}$ represents the unit direction vector of the signal incident on the array, and 〈•〉 represents the inner product operation.

The steering vector from Equation (1) is the same with the steering vector of the scalar hydrophone array, because the scalar hydrophone array can only obtain sound pressure. Considering the velocity from the two-dimensional vector hydrophone, the steering vector $a_{v}\left(\theta \right)$ of the vector hydrophone array can be expressed as follows:

(2) $$\begin{array}{c} u\left(\theta \right)={\left[\begin{array}{ccc} 1 & \cos\theta & \sin\theta \end{array}\right]}^{T}\\ a_{v}\left(\theta \right)=u\left(\theta \right)⊗a_{p}\left(\theta \right) \end{array}$$

where u(θ) represents the steering vector of the two-dimensional vector hydrophone’s response to the signal, λ is the signal wavelength, and the symbol ⊗ represents the Kronecker product operation, $a_{v}(\theta )\in C^{3M\times 1}$. Thus, the beam response of the vector hydrophone array is given by:

(3) $$p(\theta )=a_{v}{\left(\theta \right)}^{T}w$$

(4) $$w={\left[w_{P_{1}},w_{V_{x1}},w_{V_{y1}},\ldots ,w_{P_{k}},w_{V_{xm}},w_{V_{ym}},\ldots ,w_{P_{M}},w_{V_{xM}},w_{V_{yM}}\right]}^{T}$$

where $w_{P_{m}},w_{V_{xm}},w_{V_{ym}}$ corresponds to the weight coefficient of the m-th vector hydrophone.

Considering the beam response of all azimuths, an omni-directional beam pattern can be obtained. In practical applications, the azimuth is generally discretized, and the discretization processing results of the design beam pattern are as follows:

(5) p=Aw

where $p={\left[p(\theta_{1}),p(\theta_{2}),\ldots ,p(\theta_{K})\right]}^{T}$ is the designed array response vector, also called the designed beam pattern, and k is the number of sampling points of the beam pattern. We call $A={\left[a_{v}(\theta_{1}),\ldots ,a_{v}(\theta_{K})\right]}^{T}\in C^{K\times 3M}$ the array manifold matrix.

## 3. Proposed Technique

### 3.1. Introduce Compressed Sensing Method to Formation Design

The sparsity of sparse arrays bring about problems such as higher sidelobes; thus, we borrowed the idea of beam pattern synthesis [45]. The purpose of beam pattern synthesis was to obtain the beamforming weight vector so that the designed beam response would approximate the desired beam response. Conventional beam pattern synthesis methods, such as the least-mean-square criterion method, propose the following optimization methods:

(6) $$\begin{array}{cc} \underset{w}{\min} & {\left\|p_{d}(\Theta_{SL})-A(\Theta_{SL})w\right\|}_{2}\\ \mathrm{st} & A(\Theta_{ML})w=1 \end{array}$$

where $\Theta_{SL}$ is the sidelobe region and $\Theta_{ML}$ is the main lobe region of beam pattern we designed. $p_{d}$ is the desired beam pattern, also called the ideal (reference) beam pattern (response), which is the beam pattern that we designed to be fitted to. According to the requirements, we can define different desired beam pattern, and we will discuss two different desired beam patterns from the simulations experiments in Section 4. Same as Section 2, the results of the discretization process are shown in Equation (7). In general, the more discretization points, the higher design accuracy, but the greater computation, so there was a compromise between the number of discretization points and the amount of computation:

(7) $$p_{d}={\left[p_{d}(\theta_{1}),p_{d}(\theta_{2}),\ldots ,p_{d}(\theta_{K})\right]}^{T}$$

The beam pattern synthesis method only considered the beam pattern fitting problem, which made the designed beam pattern close to the desired beam pattern. This paper considered adding the objective function of the sparsity of the array elements’ number, introducing the compressed sensing method to the array optimization, and constructing a new array optimization problem, while making sparse the number of array elements and fit of the desired beam pattern.

In order to add the sparsity of the array elements’ number and obtain the optimal sparse array, we first constructed a user-chosen overcomplete array element set, which had to cover the position of the optimal array element and meet the requirements of quantification accuracy. Through the discrete sampling of the potential array elements’ positions, an overcomplete set of positions $\rho =[\rho_{1},\ldots ,\rho_{N}]$ could be obtained, in which N represented the number of position sampling points. Thus, the optimal array element position $\rho_{m}$ constituted a smaller subset of the set, which meant M<<N. In other words, the optimal location was sparse. For each element in the overcomplete position sampling set ρ, the array manifold matrix $A(\Theta )\in C^{K\times 3N}$ and weight coefficient $w\in C^{3N\times 1}$ corresponding to the potential position could be constructed.

It can be seen that the weight coefficient contained the information of the positions and multiple channels of the vector hydrophone array. Each array element channel corresponded to a weight, which meant the sparsity of the elements’ number led to the sparsity of the weights’ number. If the sparsity of the weights could be achieved, the sparsity of the elements’ number could be obtained too. By optimizing the weights, the array design could be completed. Thus, we introduced sparsity by selecting weight coefficients, and give as few non-zero weight coefficients as possible. While seeking the minimum fit error between the desired beam pattern and the designed beam pattern, we attached a sparsity penalty function constraint on the sparsity of the array optimization weights. Thus, the objective function to be sought is:

(8) $$f(w|p_{d})={\left\|p_{d}-Aw\right\|}_{2}+\eta g(w)$$

The first term ${\left\|p_{d}-Aw\right\|}_{2}$ in the Equation (8) is used to express the fitting error between the desired beam pattern $p_{d}$ and the designed beam pattern Aw. Essentially, it restricts the error between the beam pattern of the designed array and the designed beam pattern. The second term g(w) is the penalty function constraining the non-zero number of weight vector w. η represents a compromise parameter between the fitting error and the sparsity constraint, also known as the regularization factor. To achieve the objective function of non-zero weight coefficients, the penalty function is the L_0_ norm, and the objective function is equivalent to be:

(9) $$\begin{array}{cc} \underset{\hat{w}}{\min} & {\left\|w\right\|}_{0}\\ \mathrm{st} & {\left\|p_{d}-Aw\right\|}_{2}\leq \alpha \end{array}$$

where α denotes the error constraint coefficient. Existing articles [21,22] have proved that the L_1_ norm is the optimal convex approximation of the L_0_ norm. The real solution can still be obtained by replacing the L_0_ norm with the L_1_ norm. Using the L_1_ norm as the penalty function can not only ensure the sparsity constraint, but also makes the objective function a convex optimization problem, which not only avoids the risk of getting into a local minimum when solving non-convex optimization problems, but also enables the objective function to be optimized by the efficient interior point method [46].

(10) $$\begin{array}{ll} \underset{\hat{w}}{\min} & {\left\|w\right\|}_{1}\\ \mathrm{st} & {\left\|p_{d}-Aw\right\|}_{2}\leq \alpha \end{array}$$

As mentioned earlier, using the L_1_ norm as the penalty function of the sparsity constraint makes the optimization problem represented by the above equation to be a convex optimization problem, so it can be solved efficiently by using the existing convex optimization toolkit.

Since the position of the array element is obtained through discrete sampling, it is difficult to avoid the quantization errors. Thus, in the next section, we consider the off-grid array design.

### 3.2. The Theoretical Derivation of Off-Grid Developed by Taylor Series Expansion

Since the positions of the array elements were obtained through discrete sampling, the conventional method had the problem of grid division. The divided grids were all discrete, so the quantization error was unavoidable by using the conventional grid division method. In the off-grid array signal model, we no longer assumed that the optimal array element position would fall exactly on the divided grid, but would allow it to appear in any position. In this case, the set of array element positions no longer belonged to the preset overcomplete set. This section introduces Taylor series expansion to solve this problem. Linear array is the most commonly used array, for the time being, this paper only considers linear arrays design, but the same idea and method can be extended to other arrays. Here we simplify $\rho_{m}={\left[x_{m},y_{m}\right]}^{T}$ to a variable containing only distance, which is $\rho_{m}=d_{m}$. We had the following assumptions:

$d=d_{1},d_{2},\ldots ,d_{N}$ denotes a discrete set of potential array element positions, and the optimal set of array element positions is $\left\{{\tilde{d}}_{1},{\tilde{d}}_{2},\ldots ,{\tilde{d}}_{M}\right\}$. Assume that there are optimal array element positions that do not belong to d; that is, for m∈{1,2,…,M}, there exists ${\tilde{d}}_{m}\notin \{d_{1},d_{2},\ldots ,d_{N}\}$ and $d_{n_{m}}$ is assumed to be the discrete position closest to the optimal array element position.

We used first-order Taylor series expansion to approximate it linearly, as follows:

(11) $$a({\tilde{d}}_{m})\approx a(d_{n_{m}})+b(d_{n_{m}})({\tilde{d}}_{m}-d_{n_{m}})$$

where $a({\tilde{d}}_{m})$,$a(d_{n_{m}})$ is the column vector of A(Θ), and $b(d_{n_{m}})$ is the derivative of $a(d_{n_{m}})$ to d.

For simplicity, we define:

(12) $$\delta_{m}=\{\begin{array}{c} ({\tilde{d}}_{m}-d_{n_{m}}),\begin{array}{ccc} & \mathrm{for} & n_{m}=m \end{array}\\ 0\begin{array}{ccc} , & & \mathrm{otherwise} \end{array} \end{array}$$

The result obtained after vectorizing the above variables is as follows:

(13) p=(A+BΔ)w

where $B=[b(d_{1}),\ldots ,b(d_{N})]$, $\Delta =\mathrm{diag}\{\delta_{1},\ldots \delta_{N}\}$, Equation (10) can be expressed as:

(14) $$\begin{array}{ll} \underset{\hat{w}}{\min} & {\left\|w\right\|}_{1}\\ \mathrm{st} & {\left\|p_{d}-(A+B\Delta )w\right\|}_{2}\leq \alpha , \end{array}$$

### 3.3. Constructing Vector to Solve Multi-channel Synchronization Optimization Problem of Vector Hydrophone

In this section, we consider the problem introduced by the vector hydrophone array design. If the sparsity of the vector hydrophone was to be guaranteed, the three weight coefficients $w_{P_{m}},w_{V_{xm}},w_{V_{ym}}$ of each vector hydrophone needed to be minimized at the same time. The formula derived from compressed sensing in Section 3.1 could not guarantee that the three weight coefficients associated with each vector hydrophone would be minimized at the same time. The final result is that the number of optimized channels was minimal. But the number of array elements was not minimal, because the three channels per position were not optimized simultaneously. This could be well understood by assuming that each position only retained the weights corresponding to one channel and set the weights of the other channels to zero, which was equivalent to no sparsity at this position. The other two channels at this position were not utilized. In short, the compressed sensing method mentioned in Section 3.1 only optimized the sparsity of the vector hydrophone array channels, but not the vector hydrophone array elements. It did not bring real sparsity, which was contrary to the original intention of our design. Therefore, we added the constraint of simultaneous optimization of channels according to the characteristics of the vector array.

To achieve this, first consider writing the three channels of the same vector hydrophone in the form of L_2_ norm, where ${\left\|w_{n}\right\|}_{2}={\left\|\left[w_{P_{n}},w_{V_{xn}},w_{V_{yn}}\right]\right\|}_{2}$ is the weight coefficient vector for the three channels of the vector hydrophone at the n-th potential array element position. Next, define a new vector based on the form of L_2_ norm, as follows:

(15) $$\hat{w}={\left[{\left\|w_{1}\right\|}_{2},{\left\|w_{2}\right\|}_{2},\ldots {\left\|w_{N}\right\|}_{2}\right]}^{T}$$

Finally, we could get the optimization equation for the design of the vector hydrophone array:

(16) $$\begin{array}{cc} \underset{w}{\min} & {\left\|\hat{w}\right\|}_{1}\\ \mathrm{st} & {\left\|p_{d}-(A+B\Delta )w\right\|}_{2}\leq \alpha \end{array}$$

Due to the existence of bilinear variables Δw, Equation (16) was a non-convex problem; thus, it was difficult to find the global optimal solution. We used a two-step iterative optimization method to solve Equation (16). We used a two-step iterative optimization method to solve Equation (16). In the first step, fix δ and solve the following optimization problem:

(17) $$\begin{array}{ll} w^{\left(q+1\right)}= & \arg\underset{w}{\min}{\left\|\hat{w}\right\|}_{1}\\ \mathrm{st} & {\left\|p_{d}-(A+B\Delta )w\right\|}_{2}\leq \alpha \end{array}$$

Since δ is fixed, the problem is reduced to a single variable optimization problem and it is the same as Equation (10). This optimization problem can also be solved with the toolbox of convex optimization. In the second step, fix w and update δ:

(18) $$\delta^{\left(q+1\right)}=\arg\underset{\delta }{\min}{\left\|p_{d}-A\left(w^{\left(q+1\right)}\right)\right\|}_{2}$$

Repeat the above process until the convergence criterion is met. Specifically, if it reaches $\sqrt{\left\|\delta^{\left(q+1\right)}-\delta^{\left(q\right)}\right\|}\leq \beta$, then terminate our proposed algorithm, where β is the threshold of iteration termination criterion. The above algorithms can be solved by convex optimization method. The procedure of the proposed algorithm is shown in Algorithm 1.
**Algorithm 1** Procedure of the proposed algorithm.Input: $p_{d}$,A
Initialization: $\delta^{\left(0\right)}=0$
Repeat: For q = 1:Q (Q the maximum number of iterations) (1) Fix $\delta^{\left(q\right)}$, and update $w^{q}$ according to Equation (17);(2) Fix $w^{q}$, and $\mathrm{update}\;\delta^{q}$ according to Equation (18); (3) If convergent, terminate the iteration and break; otherwise, return to first step;End.  Output: w,δ


For convenience, we referred to an algorithm based on CS as the “algorithm-CS”. Similarly, we called an algorithm based on CS using the off-grid method as the “OG-−CS”. The algorithm proposed in this paper was used for vector array; thus, we can call the algorithm “V−OG−CS”.

## 4. Property Analysis

In order to verify the performance of the algorithm proposed in this paper, we used two different desired beam patterns as the fitting targets of the array design.

### 4.1. Simulation 1

A classic Dolph–Chebyshev [47] was adopted as the desired beam pattern.

We first compared the design results of the V−OG−CS algorithm and CS optimization methods for vector hydrophone arrays to verify the role of the multi-channel simultaneous optimization processing proposed in this paper. For reference, we also compared the optimized processing results of the scalar hydrophone array. The optimization method of the scalar hydrophone array was also the CS method mentioned in this paper. The number of grids used in the CS algorithm was 201, and the threshold α=0.05. The array aperture was 10 m and the half-wavelength was 0.5 m, which had 21 array elements uniformly distributed. The sidelobe level of the designed Dolph–Chebyshev was −30 dB. Table 1, Table 2 and Table 3 show the positions of the array elements and the selected channels after being optimized by the three methods. Since the array element positions of the vector hydrophone array optimized by V−OG−CS algorithm were used in all three channels, the selected channels are not indicated in the Table 3. For the iterative process of the algorithm proposed in this section, we set the maximum number Q of iterations to 20, and the threshold β of the iteration termination criterion to be 0.0001.

By comparing the array design results of the three methods, we could see that the vector hydrophone array optimized by CS did not get the optimal sparsity because the multi-channel simultaneous optimization processing was not adopted, and each array element did not fully use the information of the three channels. The vector hydrophone at each position used only one channel; thus, the sparsity was almost the same as the optimal scalar hydrophone array, while the algorithm V−OG−CS proposed in this paper designed the most sparse vector hydrophone arrays, reducing the number of vector hydrophone array elements by 50%, and greatly increasing the sparsity of the vector hydrophone array elements.

**Figure 2:**
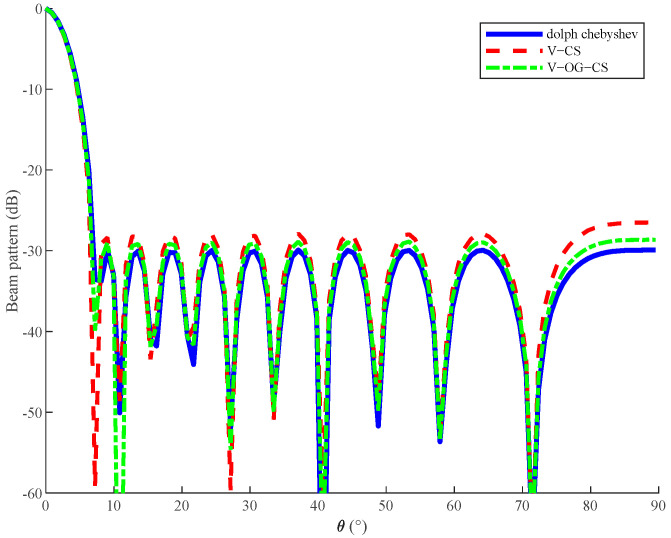
Beam patterns of different methods.

Next, we compared the beam patterns obtained by different methods to verify the fitting effect of proposed algorithm V−OG−CS. The beam patterns shown in Figure 2 were the desired response of Dolph–Chebyshev, the optimized response of the V−OG−CS algorithm, and the optimized response of the V−CS algorithm, respectively. It can be seen from the figure that the V−OG−CS algorithm proposed in this paper could provide a more accurate array response than the V−CS algorithm, and achieve the correct main lobe position and sufficient side lobe attenuation to meet the requirements.

In order to better describe the accuracy of fitting the desired beam pattern, we used the performance indicator $\varepsilon ={\left\|p_{d}-Aw\right\|}_{2}$ to describe the performance comparison between different algorithms, which was the L_2_ norm of the error between the desired beam pattern and the designed beam pattern. Since the CS method had already been constrained to have fixed parameters, we wrote only the error of the proposed algorithm V−OG−CS as follows.

Table 4 shows errors between the desired beam pattern and beam pattern of the V−OG−CS algorithm under different sidelobe level constraints. We can see that the V−OG−CS algorithm proposed in this paper could be more effective than the V−CS method under different array design parameters. It fitted the desired response better, and could meet our expected requirements. The reason why the V−OG−CS algorithm was better than CS is that the V−OG−CS algorithm used for array design added an adjustment parameter β, which made the designed array element position almost identical to the optimal array element position. As for the selection of β, we could choose the lower limit as far as possible according to actual requirements, because another convergence condition was the maximum number of iterations, except for β. As long as one of them met the termination condition, this iteration was terminated. Therefore, even if the selected value of β was small, it would not affect the convergence of the algorithm in this paper.

### 4.2. Simulation 2

In Simulation 1, it can be seen that the V−OG−CS algorithm can fit the desired beam response well with very small errors. In order to better verify the methods in this paper, we focused on the advantages and disadvantages of the design beam response obtained by the algorithm proposed in this paper. Thus, we used another desired beam pattern as the target to constrain and fit. This desired beam pattern had a primary maximum direction of 1, which meant that the target in this direction was distortionless, while the responses in the other directions were zero, which meant that all interference noise in the direction range was suppressed. The main lobe angle was 0°, a 10° transition area nearby. This desired beam pattern could not be completely fitted, but by fitting this beam pattern, we could better observe the performance of the methods proposed in this paper.

In this simulation, we added the beam pattern synthesis method of the uniform linear array mentioned in Equation (6) from Section 3.1 as a comparison. We applied the algorithm to the beam pattern synthesis of the vector hydrophone array, which could be solved by a toolbox of convex optimization (CVX), and we referred it to as V−ULA−CVX.

**Figure 3:**
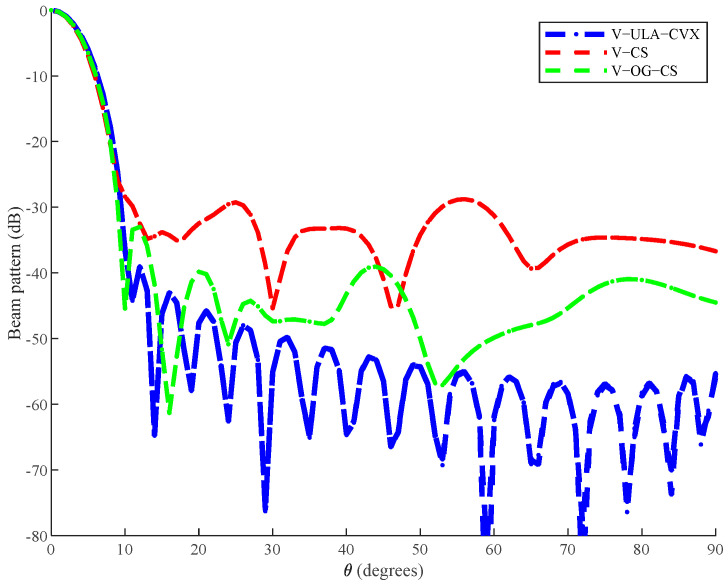
Beam patterns of different methods.

Similarly, the array aperture was 10 m, which meant the number of uniform linear array elements in this simulation was 21, and the element spacing of a uniform linear array was 0.5 m. Figure 3 shows that although the desired beam pattern could not be fully fitted, the V−OG−CS algorithm proposed in this paper still demonstrated relatively good performance and incomparable superiority. First of all, the beam pattern designed by the V−ULA−CVX method had the best performance in both the sidelobe levels and the width of the main lobe, which was our expected result, because the uniform linear array had more array elements and the optimization goal was to minimalize the error of the beam pattern. The number of array elements of V−OG−CS algorithm was 10, which was 50% less than the uniform linear array. Even so, the beam pattern obtained by V−OG−CS algorithm was basically close to the beam pattern of V−ULA−CVX and only fluctuated in the distant sidelobes, while the distant sidelobes had little effect on the array signal processing to process adjacent target resolution. In addition, the maximum sidelobe level of V−OG−CS algorithm was also close to the V−ULA−CVX method. This suggests that the V−OG−CS algorithm could achieve an expected approximation of the desired response, despite the need for fewer vector hydrophones (50% reduction).

Likewise, consistent with the results shown in Simulation 1, the V−OG−CS algorithm could bring a better fitting performance than the V−CS method, and the performance advantage embodied by the V−OG−CS algorithm was even more pronounced, with significantly lower side lobes throughout the entire azimuth, which would bring great benefits to the subsequent array signal processing.

Note that compared with Dolph–Chebyshev, the desired beam pattern used in this simulation could not be fully fitted. Therefore, the value of parameter α used in this simulation was larger than that in Simulation 1. Next, we considered the impact of α on the algorithms’ performance. Four values of parameters α were be considered, as shown in Table 5. The table summarized the performance of these two methods for these values. It can be seen that the increased value increased the allowable error, thereby allowing the additional sparsity. However, the increased value brought about the loss of beam pattern fitting at the same time. Additionally, the V−OG−CS algorithm could bring better fitting performance than the V−CS method for varying values of α.

## 5. Conclusions

In this paper, the design problem of the sparse vector hydrophone array was solved for the first time. A method based on compressed sensing was proposed to solve the array optimization problem of multi-channel vector hydrophone array. Unlike the scalar hydrophone, each vector hydrophone had three channels, corresponding to three weight coefficients, which had to be minimized at the same time to ensure a true sparse solution. Therefore, we proposed a modified L_1_ norm minimization based on compressed sensing for this problem, which realized the simultaneous optimization of multiple channels of the same vector hydrophone array element. The design simulations showed that compared with the CS algorithm which did not use the simultaneous optimization strategy in this paper, the V−OG−CS algorithm used fewer array elements (50% less than the uniform linear array), and greatly improved the sparsity of the vector hydrophone array. At the same time, the V−OG−CS algorithm could also achieve the various requirements of the array design, and reach an accurate approximation of the desired beam pattern. In addition, considering the problem of quantization errors caused by CS grid processing, this paper introduced the off-grid algorithm into the vector hydrophone array design. The design simulations also showed that V−OG−CS can fit the desired beam pattern well. Compared with the design beam pattern of the traditional CS algorithm, the proposed V−OG−CS algorithm embodied obvious performance advantages: With the lower sidelobes throughout the entire azimuth, it brought great benefits to our subsequent array signal processing. Finally, there was no restriction on the type of array beam pattern to be synthesized; thus, arbitrary array and any shape of the beam pattern could be processed. Future research can be extended to planar arrays.

## Figures and Tables

**Table 1:** Array element positions of scalar hydrophone array optimized by compressed sensing (CS).

Number	1	2	3	4	5	6	7	8	9	10	11	12	13	14	15
Position	0	0.75	1.6	2.4	3.2	4	4.45	5	5.55	6	6.8	7.6	8.4	9.25	10

**Table 2:** Array element positions of the vector hydrophone array optimized by CS.

Number	1	2	3	4	5	6	7	8	9	10	11	12	13	14
Position	0	0.8	1.65	2.45	3.25	4.05	4.8	5.2	5.95	6.75	7.55	8.35	9.2	10
Channel	$v_{x}$	$v_{x}$	$v_{y}$	$v_{y}$	p	p	$v_{y}$	$v_{x}$	$v_{x}$	p	p	$v_{x}$	$v_{y}$	$v_{y}$

**Table 3:** Array element positions of the vector hydrophone array optimized by V−OG−CS.

Number	1	2	3	4	5	6	7	8	9	10
Position	0	1.614	3.235	4.007	4.722	5.278	5.993	6.765	8.386	10

**Table 4:** Errors between the desired beam pattern and beam pattern of the V−OG−CS algorithm.

SSL	−20	−22	−24	−26	−28	−30	−32	−34	−36	−38	−40
ε	0.018	0.021	0.022	0.025	0.024	0.031	0.032	0.03	0.032	0.035	0.036

**Table 5:** Performance comparison of V−OG−CS and V−CS for different values of α.

α	ε V−OG−CS	ε V−CS	Number of Elements (V−OG− CS)	Number of Elements (V−CS)
0.3	0.25	0.3	12	12
0.4	0.31	0.4	11	11
0.5	0.39	0.5	9	9
0.6	0.48	0.59	8	8
